# Depolymerization and Nanoliposomal Encapsulation of Grape Seed Condensed Tannins: Physicochemical Characterization, Stability, In Vitro Release and Bioaccessibility

**DOI:** 10.3390/antiox14091123

**Published:** 2025-09-16

**Authors:** Carolina F. Morales, Marcela Zamorano, Natalia Brossard, Andreas Rosenkranz, Fernando A. Osorio

**Affiliations:** 1 Department of Food Science and Technology, Technological Faculty, University of Santiago (USACH), Av. El Belloto 3735, Estación Central, Santiago 9170022, Chile; carolina.moralesc@usach.cl (C.F.M.); marcela.zamorano@usach.cl (M.Z.); 2 Department of Fruit Production and Enology, School of Agricultural Science and Natural Systems, Pontificia Universidad Católica de Chile, Av. Vicuña Mackenna 4860, Macul 7820436, Chile; ndbrossa@uc.cl; 3 Department of Chemical Engineering, Biotechnology and Materials, Faculty of Physical and Mathematical Sciences, University of Chile, Av. Beauchef 851, Santiago 8370456, Chile; andreas.rosenkranz@uchile.cl

**Keywords:** nanoliposomes, condensed tannins, depolymerization, in vitro liberation, enhancement bioaccessibility

## Abstract

Condensed tannins from grape seed residues show high antioxidant activity but low oral bioavailability because of their high degree of polymerization and covalent interactions with proteins. This study aimed to improve their bioaccessibility through depolymerization and encapsulation. Depolymerization was carried out using microwave-assisted SN1 reactions with gallic acid as a nucleophile under food-grade conditions, mainly producing epicatechin monomers with 99.8% polymer degradation efficiency. Importantly, the inhibition of ABTS●+ and DPPH● radicals remained unaffected (*p* > 0.05), indicating that depolymerization preserved the antioxidants’ redox function, maintaining about 90% of their inhibition activity. The products were encapsulated in phosphatidylcholine liposomes, which had nanometric sizes and high encapsulation efficiency (83.11%), and remained stable for up to 60 days. In vitro release of nanoliposomal epicatechin in a D1 simulant was less than 10% after 48 h, fitting a Weibull model (β = 0.07), suggesting sub-diffusive transport and demonstrating high bioactive retention capacity in aqueous systems. During in vitro digestion, bioaccessibility of gallic acid and epicatechin reached 95.61 ± 0.58% and 98.56 ± 0.81%, respectively, with a 2333% increase in the bioaccessible mass of flavan-3-ols in native liposomal condensed tannins, which otherwise showed no detectable bioaccessibility. These findings highlight the potential of polyphenols from agro-industrial waste with enhanced bioaccessibility for applications in nutraceuticals and functional foods.

## 1. Introduction

Agri-food industries produce a large amount of waste during product processing, including peels, seeds, leaves, and stems, which make up a significant portion of fruit and vegetable waste [1]. Currently, this waste is mainly managed through landfills, composting, or incineration [2], even though it contains important bioactive compounds such as polyphenols and antioxidants, which have great potential for improving health and preventing various diseases [3]. Globally, it is estimated that nearly 1.3 billion tons of food are wasted each year, with fruits and vegetables accounting for around 50% of this amount [4], highlighting a valuable and untapped opportunity for their revaluation. Cancer, one of the leading causes of death worldwide, recorded 20 million new cases and 9.7 million deaths in 2022 [5]. It is estimated that by 2050, cancer cases will increase by 77%, driven by factors such as unhealthy diets and oxidative stress [6], but could be mitigated with natural antioxidants [7]. Up to 50% of cancer deaths could potentially be prevented through strategies that include natural antioxidant compounds [8].

In this context, condensed tannins (CT) from grape seeds (*Vitis vinifera* L.), a byproduct of the agro-industry that produced 930 million liters of wine in Chile in 2024 [9], emerge as a promising solution. These polymers, made up of flavan-3-ol units (mainly catechin and epicatechin), exhibit antioxidant, anti-inflammatory, and anticancer properties [10,11,12]. However, their high polymerization (>5 units) significantly limits their bioavailability, making them almost undetectable in blood [12,13]. Therefore, depolymerization methods to obtain monomers or oligomers (≤4 units) have become essential [14,15].

Various strategies have been explored to increase the bioaccessibility of condensed tannins by depolymerizing them into smaller oligomers. To achieve this, processes based on high temperatures and the incorporation of expensive nucleophiles have been used, including catechin, epicatechin, epicatechin gallate, captopril, tiopronin, and sulfite/catechin combinations [14,16,17,18,19,20]. However, many of these reactions have been carried out under conditions that are not compatible with food applications, as they typically use organic solvents such as methanol, and also lead to the formation of adducts not present in nature.

Recently, our research team developed a food-grade method for depolymerizing condensed grape seed tannins with microwave assistance, making it suitable for food, nutraceutical, and cosmetic applications. This new method eliminates the drawbacks of using toxic organic solvents and the high costs of reagents by proposing the use of low-cost nucleophiles such as gallic acid (GA) [21].

Furthermore, isolated phytochemicals are susceptible to chemical degradation when exposed to environmental stresses such as moisture, light, heat, oxygen, and pH shifts during food processing and storage, as well as during passage through the human duodenum [22].

Although fractionating condensed tannins into their monomeric units offers a partial solution to bioaccessibility challenges, these monomers can still degrade at neutral and alkaline pH, becoming unstable in the gastrointestinal tract, which could further reduce their bioaccessibility [22].

Previous studies have indicated that polyphenol stability can be enhanced using different colloidal delivery systems, such as nanoemulsions, nanogels, and nanoparticles [23,24,25].

Recent studies highlight the use of lipid-based vesicular systems such as liposomes, which have garnered significant interest as a way to enhance the delivery of bioactive molecules, such as drugs, to various organs and tissues in the human body. Their advantages as delivery systems include encapsulating both hydrophobic and hydrophilic cargoes, providing sustained release, improving bioaccessibility [26] and nutrient stability, increasing individual absorption capacity, and improving overall food quality [27]. Prior research has shown that liposome digestion and absorption in the gastrointestinal tract are complex processes, involving numerous physicochemical and biochemical reactions [28]. Consequently, the digestion behavior of liposomes can vary depending on their interfacial composition, membrane composition, interactions with the encapsulated substance, and size [29,30,31]. Size plays a crucial role in the bioavailability of the active compound, as smaller vesicles tend to enhance absorption, with the size inversely related to the absorption efficiency of the active compound [32].

Nanometric vesicles (<100 nm) have been shown to significantly enhance the uptake and absorption of the encapsulated bioactive at the target site, increasing bioaccessibility and enabling targeted intestinal release [22,31,33].

In the present study, we propose that depolymerizing condensed tannins can produce biocompatible monomers with high antioxidant capacity suitable for food-grade applications. Subsequently, liposomal nanocapsules can be fabricated to enhance their bioaccessibility. The effectiveness of this approach will be evaluated using an in vitro gastrointestinal digestion system. Previous studies on depolymerization have primarily focused on optimizing experimental conditions and identifying the resulting compounds; however, they have not addressed delivery strategies such as encapsulation or their evaluation in in vitro digestion systems [16,17,18,19,20]. Conversely, research on nanoencapsulation has not included depolymerization within its approaches [34]. To date, no studies have been reported that combine both strategies (depolymerization and nanoencapsulation), highlighting an open avenue for investigating how their integration could enhance the bioaccessibility of these antioxidant compounds.

Therefore, depolymerizing condensed tannins into their monomeric units could facilitate the formation of nanoliposomes. In conclusion, it is essential to first understand the effect of chemical depolymerization of condensed tannins on their average degree of polymerization, and how this impacts their antioxidant capacity, final liposome size, release kinetics, and behavior in gastrointestinal digestion, before developing real-world food applications. Importantly, these physicochemical changes can translate into functional benefits in food systems, such as extended shelf life through delayed oxidative degradation, improved sensory stability by reducing undesirable astringency, and more consistent release of bioactive compounds during storage and consumption [27].

## 2. Materials and Methods

### 2.1. Chemicals and Reagents

Grape seed condensed tannins were obtained from Laffort (Bordeaux, France). GA for synthesis was sourced from Merck (Darmstadt, Germany). Folin–Ciocalteu phenol reagent, gallic acid for synthesis (GA), potassium chloride (KCl), calcium chloride (CaCl_2_), potassium phosphate (KH_2_PO_4_), sodium chloride (NaCl), and ammonium carbonate ((NH_4_)_2_CO_3_) were purchased from Merck (Darmstadt, Germany). Sodium hydroxide reagent grade ≥ 98% (NaOH) pellets (anhydrous), sodium acetate, acetic acid, 2,2′-azino-bis-3-ethylbenzothiazoline-6-sulfonic acid diammonium salt (ABTS•+), and 2,2-diphenyl-1-picrylhydrazyl (DPPH•) were obtained from Sigma-Aldrich (Steinheim, Germany). Analytical standards of (+)-catechin and (−)-epicatechin, primary reference standards of epicatechin gallate and catechin gallate (purity ≥ 99%), L-α-Lecithin soybean (phosphatidylcholine purity ≥ 97%), glycerol, and digestive enzymes (pepsin, bovine bile, and intestinal lipase) were obtained from Sigma-Aldrich. Ethanol absolute (no denatured, purity ≥ 99.5%) was sourced from Merck KGaA (Darmstadt, Germany). Hydrochloric acid was purchased from Merck (Darmstadt, Germany). Milli-Q water (Elga Purelab system) and deionized water were used as solvents. Citric acid and sodium citrate were obtained from Winkler (Santiago, Chile).

### 2.2. Extraction and Characterization of Depolymerized Grape Seed Condensed Tannins

#### 2.2.1. Chemical Depolymerization of Grape Seed Condensed Tannins

The mean degree of polymerization (mDP) of grape seed condensed tannins (CT) was determined using the standardized protocol developed by Bianchi et al. (2016) [35].

Depolymerization was performed through unimolecular nucleophilic substitution (SN1) reactions using gallic acid as the nucleophile, which follows second-order kinetics [21]. Ten 10 mg/mL of grape seed condensed tannins (CT), with an average degree of polymerization of 8, was dissolved in 80% *v*/*v* aqueous ethanol with hydrochloric acid (HCl) at pH 0.5 and dispersed by sonication for 2 min. Subsequently, 5.88 mg/mL of gallic acid (GA) was added, and the mixture was incubated in a PTFE vessel for microwave synthesis at 60 °C for 60 min. The reaction was halted by immediately placing the vessel in a water/ice bath and adding 5 N NaOH until the pH reached 3, resulting in suspensions of depolymerized grape seed tannins (STD). The final suspensions of depolymerized condensed tannins were concentrated and subsequently lyophilized for preservation (Freeze dryer: Büchi L-200, Surat, India; vacuum pump: PFEIFFER Duo 6M, Germany). Since the samples did not contain biological systems or vesicular structures, the use of a cryoprotectant was not required. The lyophilization process was conducted after pre-freezing at –80 °C, followed by primary drying at 1 mbar and –55 °C, and a secondary drying phase until a residual moisture content of <1% was achieved. Finally, the samples were stored at −20 °C, producing a dry powder of antioxidants (TD), which were redispersed for subsequent analysis. The efficiency of the depolymerization was assessed by ultra-high-performance liquid chromatography (UHPLC), as described in Section 2.2.2, and was calculated using Equation (1).

(1) $$RY\left(\%\right)=\frac{P_{i}\left(mg\right)-P_{R}(mg)}{P_{i}\left(mg\right)}*100$$

where RY is the percentage of reaction yield (%); *P_i_* is the mass of added CT (mg); and *P_R_* is the mass of remaining CT (mg).

#### 2.2.2. Chromatographic Analysis Using Ultra-High-Performance Liquid Chromatography (UHPLC)

Samples were analyzed using reverse-phase ultra-high-performance liquid chromatography (UHPLC; Thermo Scientific Dionex UltiMate 3000, MA, USA). They were filtered through a 0.22 μm PTFE filter and separated on a C18 column (5 μm, 4.6 × 150 mm, Thermo Scientific Waltham, MA, USA). The system includes a quaternary pump, an autosampler, a column thermostat, a UV detector, and a photodiode array detector (DAD).

To quantify the condensed tannins and reaction products, the chromatographic conditions were as follows: an injection volume of 10 μL, a flow rate of 1 mL/min, and a column temperature of 30 °C. The mobile phase included phase A, trifluoroacetic acid/water (0.1:100 *v*:*v*), and phase B, water/acetonitrile (1:4 *v*/*v*) containing 0.08% trifluoroacetic acid. Polyphenols were detected at 280 and 545 nm. Peaks were integrated into the chromatograms, and the external standard method (catechin, epicatechin, gallic acid, catechin gallate, epicatechin gallate, condensed tannins) was used to determine the concentrations of the reaction products [35].

#### 2.2.3. Structural Free Radical Scavenging Activity

ABTS•+ (2,2′-azino-di-[3-ethylbenzothiazoline sulfonate]) and DPPH• (2,2-diphenyl-1-picrylhydrazyl) radical scavenging capacities were measured using absorbance, expressed as the percentage inhibition of both radicals with Equations (2) and (3), respectively [21].

(2) $$\%ABTS{•}^{+}\;or\;\%\mathrm{DPPH}•\;inhibition=\frac{A_{control}-A_{mixture}}{A_{control}}*100$$

where $A_{control}$: absorbance of the mixture of ethanol and *ABTS•^+^* solution at 734 nm or *DPPH•* at 517 nm; $A_{mixture}$: absorbance of the sample solution mixture.

#### 2.2.4. Total Polyphenol Content (TPC)

TPC was determined using the Folin–Ciocalteu method [36], which involves the Folin–Ciocalteu reagent and measuring absorbance at 760 nm with a spectrophotometer. The result is expressed as the total phenol content equivalent to gallic acid per gram of dry weight polyphenols (GAeq mg/g).

### 2.3. Obtaining and Characterizing Nanoliposomal Suspensions That Encapsulate Depolymerized Grape Seed Tannins (LTD)

#### 2.3.1. Preparation of Nanoliposomal Suspensions

Nanoliposomal suspensions with depolymerized condensed grape seed tannins (LTD) were prepared through a heating and homogenization process, with lamellarity and size reduction achieved by ultrasound cycling [37,38], with some modifications. Depolymerized grape seed condensed tannins (STD) (1 mg/mL) and L-α-Lecithin Soybean (PC) (1 mg/mL) were dissolved in ethanol-citrate buffer (0.1 M at pH 3) at 60 °C for 1 h. Then, 0.76 g of glycerol was added, followed by a second heating at 60 °C for 1 h. Subsequently, 5 vortex cycles and 10 ultrasound cycles (HIELSCHER UP100H, Heidelberg, Germany, max. 100 W) were applied at 90% amplitude and 30 kHz, and 10 ultrasound cycles were performed, consisting of 1 min of sounds followed by 1 min resting. The samples were maintained in amber jars submerged in a water bath to avoid overheating, while the temperature was monitored during each rest interval, ensuring that no case exceeded 60 °C.

A liposome suspension containing grape seed condensed tannins (LT) was prepared and used as a control system.

#### 2.3.2. Encapsulation Efficiency (%EE) and Loaded Capacity (%LC)

To determine the encapsulation efficiency and loading capacity of LTD, the samples were centrifuged at 21,060× *g* for 60 min at 4 °C [39] using an ultracentrifuge (Hanil Scientific Inc. Supra R22, Gimpo, Republic of Korea).

The supernatant was then analyzed with ultra-high-performance liquid chromatography (UHPLC) (Thermo Fisher Scientific Ultimate 3000, Waltham, MA, USA), as described in Section 2.2.2. The %EE was calculated using Equation (3), and the %LC was calculated using Equation (4) [40].

(3) $$EE\left(\%\right)=\frac{{\left[TD\right]}_{i}-{\left[TD\right]}_{L}}{{\left[TD\right]}_{i}}*100$$

(4) $$LC\left(\%\right)=\frac{{\left[TD\right]}_{i}-{\left[TD\right]}_{L}}{{\left([TD\right]}_{i}-{\left[TD\right]}_{L})+[PC]}*100$$

where ${\left[TD\right]}_{i}$ is the initial concentration of STD in the liposomal suspension; ${\left[TD\right]}_{L}$ is the concentration of free STD in the medium; and [*PC*] is the initial concentration of L-α-Lecithin Soybean.

#### 2.3.3. Hydrodynamic Particle Diameter, Polydispersity Index, and Zeta Potential

The hydrodynamic mean particle diameter (HPD), polydispersity index (IPD), and zeta potential (ζ) were measured using a dynamic light scattering instrument (Litesizer 500, Anton Paar). The zeta potential was calculated with the Smoluchowski model at a diffraction angle of 173° [25]. The suspensions were diluted with an appropriate buffer (sodium acetate and acetic acid buffer solution: 0.1 M; pH 3.0 ± 0.1) before analysis to prevent multiple dispersion effects.

#### 2.3.4. Morphological Properties and Microstructure

The microstructure of LTD was examined using transmission electron microscopy (TEM) on a Talos F200C G2 microscope (Thermo Scientific, Waltham, MA, USA), using the negative staining technique and uracil acetate [41]. Samples were placed on a Formvar-coated carbon copper grid (300 mesh, 3 mm diameter, HF 36). A 2% *w*/*v* uranyl acetate solution was then added to the samples and left to stand for 1 min at 25 °C. Finally, the grids were air-dried in an oven (model LDO-150F, LabTech, Gwangju-si, Republic of Korea) at 25 °C for 10 min.

#### 2.3.5. Rheological Properties

For the evaluation of the rheological behavior of LTD, a rheometer (Discovery Hybrid Rheometer HR2, TA Instruments, New Castle, DE, USA) with a truncated cone and plate setup (1.008° angle, 60 mm diameter, and 27 μm gap between the geometry and the plate) was used. On the lower plate, which was equipped with a Peltier temperature control system, 1.5 mL of the sample was placed, and the upper plate was lowered until it was 27 μm above the lower plate, then allowed to stabilize for 5 min at the initial temperature of 20 °C. Oscillatory amplitude tests were performed to identify the linear viscoelasticity range, using a strain from 0.1% to 30%. Afterwards, temperature sweeps ascending from 4 °C to 85 °C and descending from 85 °C to 4 °C were conducted at a rate of 2 °C/min, with deformations of 0.3% (LT) and 1.0% (LTD), and an angular frequency of 1 Hz, to study the suspension’s behavior in response to temperature changes [24].

#### 2.3.6. LTD Suspension Stability

The long-term stability of the LTD suspensions was examined by monitoring changes in CAO (DPPH• and ABTS•+ assays), TPC, %EE, DHP, and PZ using the methodologies outlined in Section 2.2.3, Section 2.2.4, Section 2.3.2, and Section 2.3.3, respectively. These parameters were measured during storage, with suspensions kept in amber glass bottles at 4 °C, at multiple time points over 60 days.

### 2.4. In Vitro Release of Nanoliposomal Suspensions Encapsulating Depolymerized Seed Tannins (LTD) in a Food Simulant

In vitro release of LTD was conducted following EU regulation No. 10/2011 using a food simulant D1 (ethanol 50%) [42], which is suitable for lipophilic foods such as milk and dairy beverages. This simulant was chosen based on guidelines and our research on LTD’s potential use in dairy and fatty products. For this, 2 mL of the LTD sample was placed into a dialysis bag made of cellulose membrane with a molecular weight cut-off of 14,000 Da. The dialysis bag was then immersed in 200 mL of the simulated medium and incubated in the dark with gentle shaking in a shaker incubator (JSSI-100C JSR, Gongju-City, Republic of Korea) at 37 °C, 170 rpm for 48 h.

Samples of 2 mL from the external medium were collected at various time points, and an equal volume of fresh medium was added to maintain the system [41,43].

In vitro LTD release studies were compared using the ST, STD, and LT suspensions; all experiments were performed in triplicate. Afterwards, in vitro release was measured with reverse-phase ultra-high-performance liquid chromatography (UHPLC; Thermo Scientific Dionex UltiMate 3000), as described in Section 2.2.2, by calculating the percentage of cumulative release (CR) with Equation (5).

(5) $$CR\left(\%\right)=\left(\frac{\left[LT\right]*f*VL}{LTtotal}\right)*100$$

where $\left[LT\right]$ is the concentration of TD in the release medium (µg/mL); f is the dilution factor; VL is the volume of the release medium (mL); and LTtotal is the total TD (µg).

Release kinetics were analyzed by plotting the cumulative release rate (*CR/CR∞*) over time. Model fit was verified using the coefficient of determination (R2) with CurveExpert Professional 2.6.5 software. The curves were fitted with the mathematical models Korsmeyer–Peppas, Higuchi, and Weibull, using Equations (6), (7), and (8), respectively [44,45].

(6) $$\frac{{CR}_{t}}{{CR}_{\infty }}=K_{R}\times t^{n}$$

where ${CR}_{t}$ is the fraction of TD released in the food simulator over time t (h), ${CR}_{\infty }$ is the maximum releasable (equilibrium) amount of TD in the simulant medium, $K_{R}$ is the release rate constant, and n indicates the release mechanism of antioxidants from LTD to the simulant. This model allows us to infer the mechanism since *n* = 0.43: Fickian diffusion (transport by diffusion); 0.43 < *n* < 0.85: anomalous transport (diffusion + relaxation); *n* = 0.85: transport type case II (relaxation or swelling); and *n* > 0.85: transport type super case II [46].

(7) $$\frac{{CR}_{t}}{{CR}_{\infty }}=k_{H}t^{1/2}$$

where $k_{H}$ is the Higuchi constant, which depends on factors such as the initial concentration, the diffusivity of the antioxidants, and the thickness of the matrix form [47].

(8) $$\frac{{CR}_{t}}{{CR}_{\infty }}=1-e^{\left(-{\left(\frac{t}{\alpha }\right)}^{\beta }\right)}$$

where *α* is scale parameter (related to the release rate), and *β*: shape parameter (related to the release mechanism), since, if *β* = 1: release of simple exponential order; *β* < 1: faster release at the beginning, then slower (restricted diffusion); and *β* > 1: slow release at the beginning and faster later (suggests complex mechanisms such as matrix erosion or swelling) [48].

### 2.5. Bioaccessibility of Liposomal Suspensions Encapsulating Depolymerized Grape Seed Condensed Tannins (LTD)

LTD and LT suspensions were subjected to standardized statical in vitro gastrointestinal digestion following INFOGEST [49], which simulates oral, gastric, and intestinal phases. For the oral phase, 4.975 mL of simulated salivary fluid and 25 µL of 0.3 M CaCl_2_ were added to 5 mL of fresh LTD. Since none of the samples contained amylase-sensitive polysaccharides, no amylases were included [50]. The mixture was adjusted to pH 7.0 and incubated in a dark, compact shaking incubator (JSSI-100C JSR, Republic of Korea) at 37 °C and 170 rpm for 2 min. The bolus after the oral phase was then mixed with 8 mL of simulated gastric fluid, pepsin (2000 U/mL), and 0.1 M HCl to reach pH 2.0, with 5 µL of 0.3 M CaCl_2_ and distilled water added to make a total volume of 20 mL. This mixture was incubated at 37 °C with continuous shaking at 170 rpm for 120 min. Next, 20 mL of gastric chyme was combined with 16 mL of simulated intestinal fluid, 40 µL of 0.3 M CaCl_2_, NaOH (1 M) to adjust pH to 7.0, bile extract (10 mM), and intestinal lipase (2000 U/mL) to simulate the duodenal phase. To measure the release of TD after each phase—oral, gastric, and duodenal—the digested samples were centrifuged at 21,060× *g* for 30 min at 4 °C in an ultracentrifuge (Hanil Scientific Inc. Supra R22, Gimpo, Republic of Korea). The aqueous phase was then analyzed using ultra-high-performance liquid chromatography (UHPLC) (Thermo Fisher Scientific Ultimate 3000, Waltham, MA, USA), as detailed in Section 2.2.2. LT samples served as controls and followed the same methodology. The bioaccessibility of LTD was calculated using Equation (9) [30]. For this study, three independent biological samples were considered, whose bioaccessibility results are expressed as the average ± the standard deviation (SD).

(9) $$bioaccessibility\left(\%\right)=(\frac{M_{ti}}{M_{0}})*100$$

where $M_{ti}$ is the total intestinal mass of an individual compound (AG, Ep, and CT) at different times during the duodenal stage, and $M_{0}$ is the initial mass of an individual compound (AG, Ep, and CT) encapsulated in LT or LTD in the fresh sample before starting the digestion process.

To examine the release of antioxidants at different times during in vitro digestion, a parallel digestion experiment with stepwise sampling was conducted. Five Erlenmeyer flasks were prepared identically, each serving as an independent replica of the in vitro digestive system. In all cases, the simulated oral phase was initiated, followed by the sequential addition of the gastric and duodenal phases. Sampling was performed by fully stopping enzymatic activity through pH adjustment, then freezing each flask at −80 °C (Thermo Scientific Forma 700 Series Ultra-Low Chest Freezer, Waltham, MA, USA) at predetermined times, allowing for independent analysis at each stage.

### 2.6. Statistical Analysis

Where applicable, all statistical analyses were performed using STATGRAPHICS Centurion XVI v.16.1.03 software (StatPoint Technologies, Inc., Warrenton, VA, USA). Results are presented as mean values ± standard error. Differences were assessed with ANOVA. The confidence level was set at 95% (*p* < 0.05). The model’s fit was verified using the coefficient of determination (R2), standard deviation, and residual analysis.

## 3. Results

The results of this study are presented below.

### 3.1. Results of Obtaining and Characterizing Depolymerized Grape Seed Condensed Tannins

The microwave-assisted acid depolymerization of condensed grape seed tannins, performed with gallic acid as the nucleophile in 80% aqueous ethanol, achieved a conversion yield of 99.9% (Equation (1)). Table 1 shows the physicochemical properties of condensed grape seed tannins before (ST) and after (STD) depolymerization using gallic acid as the nucleophile under microwave-assisted acidic conditions.

### 3.2. Results of Obtaining and Characterizing Nanoliposomal Suspensions Encapsulated Depolymerized Condensed Tannins

#### 3.2.1. Results of Physicochemical Parameters of Liposomal Suspensions

Table 2 shows the key physicochemical parameters of liposomal suspensions LT containing untreated condensed tannins (mainly CT) and nanoliposomal suspensions LTD encapsulating depolymerized condensed tannins (epicatechin as the main depolymerization product).

#### 3.2.2. Results of Morphological Properties and Microstructure

**Figure 1:**
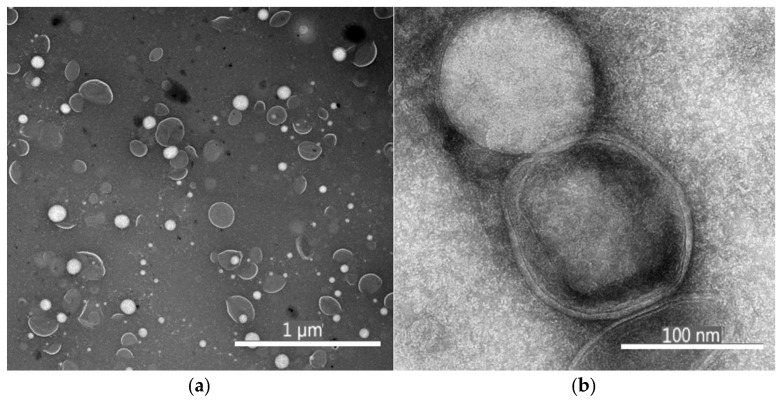
Transmission electron micrographs (TEM) of LTD with magnification (**a**) 6700× and pixel size 4.369 nm and (**b**) image with 110,000× magnification and pixel size 0.266 nm.

The formation of depolymerized condensed tannin liposomes was confirmed by transmission electron micrographs (TEM), which showed vesicles between 80 and 120 nm (Figure 1). 

#### 3.2.3. Results of the Rheological Behavior of Liposomal Suspensions

Oscillatory amplitude tests were conducted to determine the linear viscoelastic range of the sample, spanning 0.1% to 30% strain at 1 Hz and 20 °C. It was found that the linear zone corresponds to deformations of 0.3% (LT) and 1.0% (LTD).

**Figure 2:**
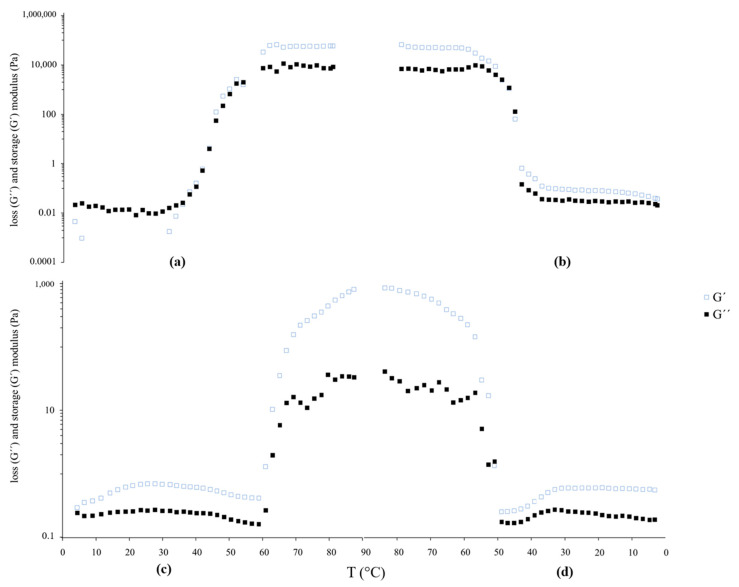
(**a**) Heating and (**b**) cooling temperature ramp profiles of LT suspensions, and (**c**) heating and (**d**) cooling temperature ramp profiles of LTD suspensions showing the evolution of storage modulus (G′) and loss modulus (G″) between 4 °C and 85 °C.

Figure 2 displays the rheological behavior of the LT and LTD suspensions.

#### 3.2.4. Results of Long-Term Stability of Liposomal Suspensions and Antioxidant Activity

**Figure 3:**
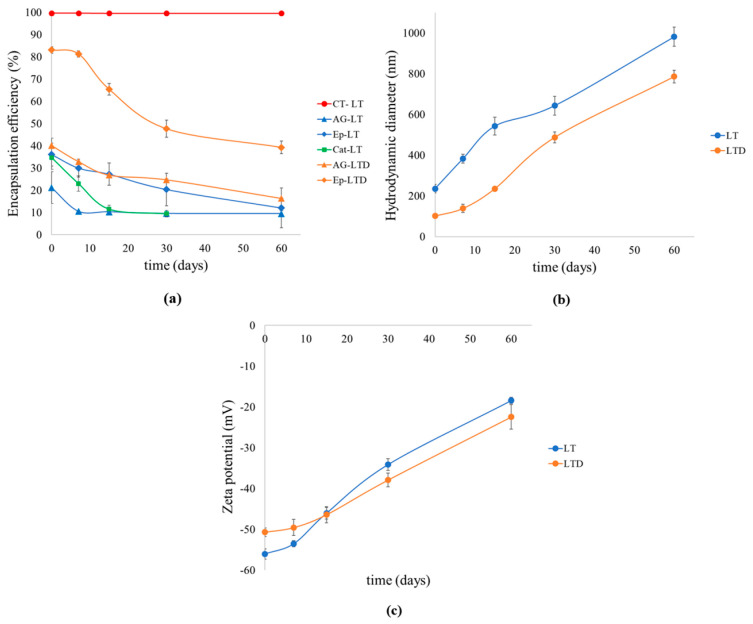
Physicochemical stability plots of liposomal suspensions of condensed tannins (LT) and depolymerized condensed tannins (LTD) during storage at 4 °C over 60 days. (**a**) Encapsulation efficiency of CT, AG, and Ep in LT, and of AG and Ep in LTD. (**b**) Changes in hydrodynamic particle diameter (nm) for LT and LTD. (**c**) Changes in zeta potential (ζ) (mV) for LT and LTD.

The stability of the liposomal systems was evaluated over 60 days under dark storage conditions at 4 °C; the results are shown in Figure 3. Values correspond to the mean ± standard deviation (*n* = 3).

**Figure 4:**
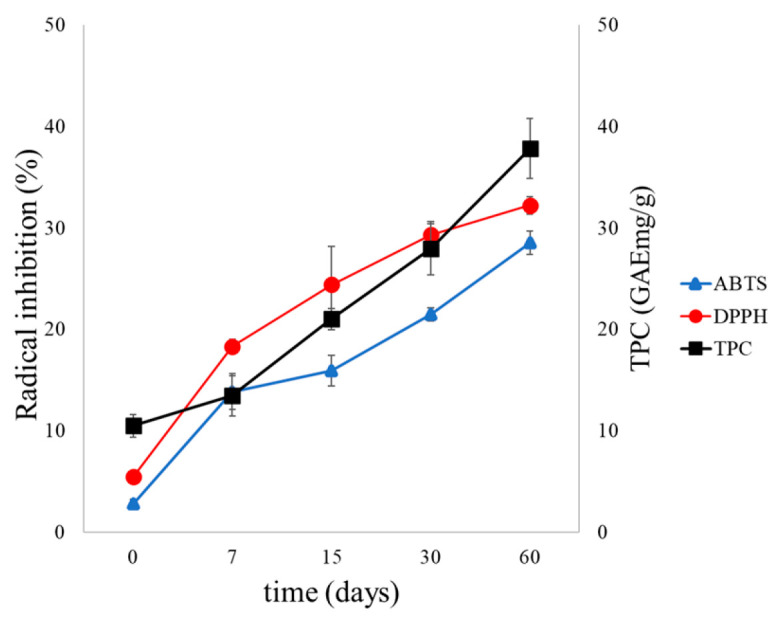
Changes in antioxidant activity (ABTS•+ and DPPH• radical inhibition percentages) and TPC plots of depolymerized condensed tannins (LTD) during storage at 4 °C over 60 days.

The antioxidant activity and TPC of the LTD systems were evaluated over 60 days under dark storage conditions at 4 °C; the results are shown in Figure 4. Values correspond to the mean ± standard deviation (*n* = 3).

### 3.3. Results of in Vitro Release Testing of Liposomal Suspensions in a Food Simulant

**Figure 5:**
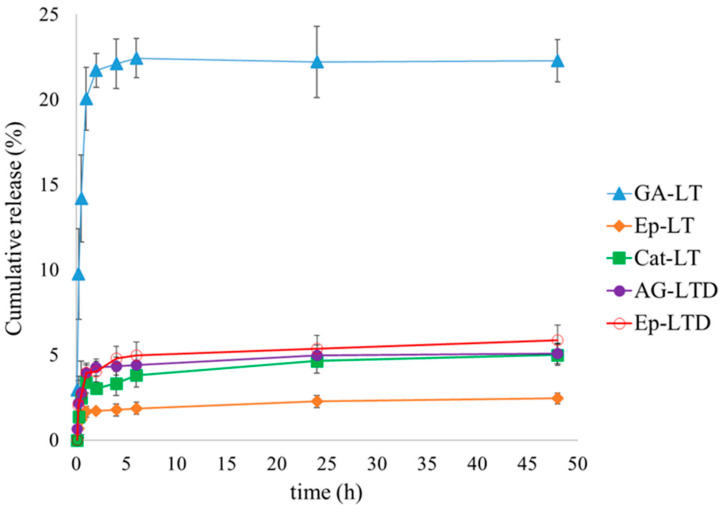
Cumulative release graph over time for antioxidant species under release conditions at 37 °C and 170 rpm in food simulant D1, suspensions of liposomal encapsulating condensed tannins (LT), and nanoliposomal encapsulating depolymerized condensed tannins (LTD).

In vitro release studies were conducted in a D1 food simulant for the LT and LTD samples at 37 °C, pH 3, and 170 rpm for 48 h. The results are shown in Figure 5. Values correspond to the mean ± standard deviation (*n* = 3).

Mathematical adjustments of the results of the in vitro release studies were conducted in a D1 food simulant for the LT and LTD samples at 37 °C for 48 h, and are presented in Table 3. Values correspond to the mean ± standard deviation (*n* = 3).

### 3.4. Results of Bioaccessibility and Digestion Release of Liposomal Suspensions

**Figure 6:**
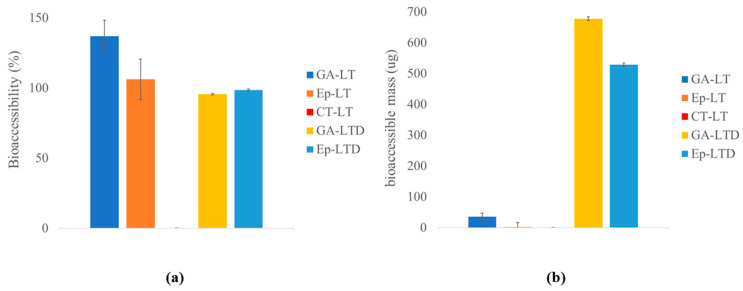
(**a**) Bioaccessibility (%) and (**b**) bioaccessible mass (µg) of gallic acid (GA), epicatechin (Ep), and condensed tannins (CT) in LT and LTD formulations after in vitro gastrointestinal digestion using the INFOGEST protocol.

Static in vitro digestion studies were performed using the INFOGEST protocol for the LT and LTD samples. The bioaccessibility data and accessible mass are shown in Figure 6. Values correspond to the mean ± standard deviation (*n* = 3).

**Figure 7:**
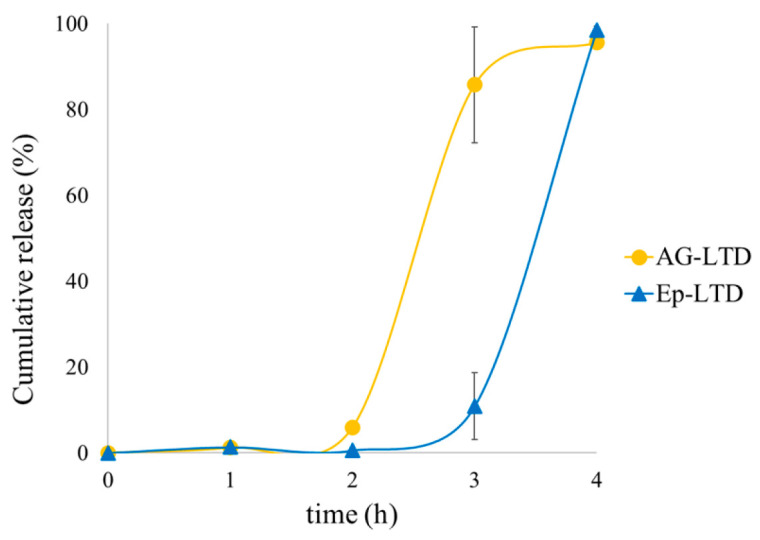
Cumulative release of AG-LTD and Ep-LTD during in vitro digestion. Time zero marks the beginning of the oral phase, up to 2 h corresponds to the gastric phase, and from 2 to 4 h indicates the duodenal phase.

The results of cumulative release for LTD systems during in vitro digestion are presented in Figure 7. Values correspond to the mean ± standard deviation (*n* = 3).

## 4. Discussion

### 4.1. Discussion of Obtaining and Characterizing Depolymerized Grape Seed Condensed Tannins

Table 1 shows the microwave-assisted acid depolymerization of condensed grape seed tannins, performed with gallic acid as the nucleophile in 80% aqueous ethanol, resulting in a conversion yield of 99.9% (Equation (1)). The total phenolic content (TPC) was significantly higher in the depolymerized extracts (TD: 287.4 ± 7.96 mg/g) compared to the original extracts (TD: 241.7 ± 13.2 mg/g), with *p* < 0.05. This increase in TPC can be linked to the exposure of phenolic groups previously hidden within the polymeric structure. Despite this structural change, the antioxidant capacity (measured by ABTS●+ and DPPH● assays) remained unchanged (*p* > 0.05), indicating that depolymerization did not diminish the antioxidants’ redox functionality, which stayed around 90% [21] . The hydrodynamic diameter decreased significantly after the reaction (from 16,860 nm to 6.2 nm), indicating effective cleavage of high molecular weight polymers into smaller, less aggregated species with increased mobility and chemical availability, confirming the efficiency of this SN1 method for producing monomers from highly polymeric tannins [21].

### 4.2. Discussion of Obtaining and Characterizing Nanoliposomal Suspensions Encapsulating Depolymerized Condensed Tannins

#### 4.2.1. Physicochemical Parameters of LT and LTD

Table 2 presents the results of the physicochemical parameters for the polymeric extracts (ST) and depolymerized products (STD with epicatechin as the main product), which were encapsulated in liposomal suspensions (LT) and nanoliposomal suspensions (LTD), respectively. The encapsulation efficiency (EE%) was significantly higher in LT (99.65 ± 0.01%) compared to LTD (83.11 ± 1.46%; *p* < 0.05). This decrease in LTD likely results from the smaller size and molecular weight of the depolymerized compounds, allowing greater diffusivity into the extravesicular medium during or after liposome formation [51]. However, the loading capacity (LC) remained high in both systems (>97%), indicating a strong affinity between the liposomal matrix and polyphenolic compounds, even after depolymerization [40].

The mean hydrodynamic particle diameter (HPD) significantly decreased from 235.3 ± 21.4 nm in LT to 101.3 ± 7.8 nm in LTD, confirming the successful formation of nanoliposomes [52]. This size reduction likely results from decreased aggregation behavior of the monomeric flavan-3-ols, which promotes the formation of smaller vesicles during ultrasonication, consistent with the HPD results reported in Table 1. The zeta potential (ζ) was negative in both formulations, measuring −56.0 ± 1.3 mV for LT and −50.7 ± 1.1 mV for LTD, indicating high colloidal stability due to electrostatic repulsion. These values correspond with the presence of phosphate groups in phosphatidylcholine, ionizable phenolic groups in the encapsulated polyphenols, and ionic species in the medium. These results demonstrate high colloidal stability caused by electrostatic repulsion [53].

The polydispersity index (PDI) was comparable between the formulations (0.24–0.26), indicating an acceptable size distribution without excessive heterogeneity [24].

Overall, these results confirmed that the depolymerization strategy influences the entire architecture of the liposomal system. The LTD formulation achieved nanometric particle size while maintaining high loading capacity and stability, providing higher surface-to-volume ratios, improving solubility, stability, bioavailability, and precise targeting, and reducing the toxicity of vesicular systems [54].

#### 4.2.2. Morphological Properties and Microstructure of LTD

Figure 1 shows the transmission electron micrographs (TEM) of LTD, confirming the successful formation of well-defined, spherical vesicles without aggregates. At higher magnification, unilamellar structures are observed, characterized by a single bilayer surrounding the aqueous core, typical of liposomes formed by ultrasonication [55]. Vesicle diameters ranged from 88 to 110 nm; these results align with those of Xu et al. (2021) [56], who prepared soybean-α-phosphatidylcholine liposomes encapsulating DHA and anthocyanins, measuring 132.2 ± 6.3 nm, and Toro-Uribe et al. [57], who produced nanoliposomes between 73.9 and 84.3 nm for encapsulating theobromine, caffeine, catechin, epicatechin, and cocoa extract. These findings are consistent with the DLS data (HPD ≈ 100 nm and uniform PDI) reported in Table 2.

#### 4.2.3. Rheological Behavior of LT and LTD

The viscoelastic behavior of the LT and LTD liposomal suspensions was evaluated using thermal sweep rheological tests over a temperature range of 4 °C to 85 °C. Figure 2 shows the changes in the storage modulus (G′) and loss modulus (G″) as temperature varies.

The LTD suspensions exhibited distinctive viscoelastic properties that suggest greater structural stability compared to traditional liposomal systems. During heating, the storage modulus (G′) consistently remained above the loss modulus (G″) throughout the entire temperature range (Figure 2c,d), indicating a primarily elastic gel-like behavior [58].

The tests also identified the phase transition temperature (Tm), which is the point where the rheological moduli change suddenly, indicating the transition of the lipid bilayer from an ordered gel phase to a crystalline liquid phase [59]. In this case, LTD showed a significant increase in Tm, reaching 57.28 °C, compared to the 37.55 °C observed for LT (Figure 2a).

This rise in Tm can be attributed to increased vesicular membrane rigidity, stemming from specific noncovalent interactions between flavan-3-ol monomers and phospholipids, including hydrogen bonds, aromatic–aromatic interactions, and associations between phenolic groups and the polar heads of phosphatidylcholine. These interactions strengthen lipid packing, reduce local fluidity, and stabilize the bilayer, which requires more thermal energy to trigger the phase transition [34,60].

Additionally, the insertion of epicatechin between the acyl tails helps stiffen the membrane, unlike the LT system, which is known for high fluidity and lateral mobility of lipid chains without components that restrict its movement [24].

The absence of a G′–G″ crossover point during LTD heating (Figure 2c) indicates that its lipid bilayer retains its structural integrity even at 85 °C. Overall, these results demonstrate that tannin depolymerization gives liposomal systems unique functional properties that cannot be achieved by polymeric tannins, suggesting a system capable of protecting antioxidant compounds under physiological conditions [61].

### 4.3. Discussion of Liposomal Suspensions’ Long-Term Stability and Antioxidant Activity

Long-term evaluation (60 days) of LT and LTD liposomal suspensions (Figure 3) reveals distinct patterns in the retention and release of phenolic compounds, driven by their molecular nature and the structure of the encapsulation system. Figure 3a shows an exceptionally stable encapsulation efficiency (%EE) for polymers (>99%), due to their high molecular weight and limited diffusion mobility, which restricts their migration through the lipid bilayer, confirming the long-term structural integrity of the vesicles [37,55]. In contrast, monomers such as catechin, epicatechin, and gallic acid exhibited a gradual decrease in %EE for both LT and LTD systems, with their retention dropping to about 50% of the initial value in both cases. This difference suggests a size-dependent passive release mechanism, where the smaller size of the monomers helps them diffuse through the lipid membrane [62] but improves bioaccessibility by decreasing the average degree of polymerization (only monomers and oligomers < 5 units are bioavailable) and by avoiding the formation of large complexes [12].

The higher retention observed in nanoliposomes (LTD) compared to larger liposomes (LT) highlights the important role of nanoarchitecture in system stability. While LTs experienced significant vesicle growth (~900 nm) (Figure 3b), LTDs remained smaller than 600 nm. This can be explained by collisions and the eventual membrane fusion of two or more liposomes caused by the random (Brownian) motion of vesicles in solution [63]. Overall, nanosized liposomes tend to be more stable than larger ones because of their lower collision frequency, higher membrane curvature, and more uniform size distribution, which reduces the chances of aggregation, fusion, or destabilization, especially under physiological or storage conditions [64,65,66]. This size stability correlates with the maintenance of highly negative zeta potential values (–30 to –50 mV) (Figure 3c), indicating sufficient electrostatic repulsion to prevent coalescence [67]. The simultaneous increase in antioxidant activity and phenolic content in the external LTD medium (Figure 4 and Figure S2) suggests that the release, although inevitable, does not compromise the functionality of the compounds. These factors also help maintain system stability by reducing the chemical destabilization of liposomes, mainly lipid oxidation and hydrolysis [68].

It is suggested that passive release may be driven more by diffusion through transient defects in the bilayer rather than by structural degradation [69,70]. Overall, these findings show that nanoliposomal formulations with monomeric flavan-3-ol antioxidants (LTDs) offer improved physicochemical stability under cold storage conditions, including better particle size, surface charge, and encapsulation capacity, as well as enhanced preservation of antioxidant functionality [71].

### 4.4. Discussion of in Vitro Release of Liposomal Suspensions in a Food Simulant

The total release of flavan-3-ols from free suspensions (ST and STD), liposomal (LT), and nanoliposomal (LTD) suspensions was measured in food simulant D1 at 37 °C for 48 h (Figure 5 and Figure S2). Both liposomal formulations showed minimal release of encapsulated compounds, with values below 5% even after 48 h for the LTD suspensions (GA and Ep). In contrast, the LT suspensions exhibited a higher release percentage, especially for gallic acid (GA), which reached about 22% at the end of the testing period. Meanwhile, the non-encapsulated formulations (ST and STD, Figure S1) displayed significantly higher release levels, up to 90%. The Higuchi model showed a low fit across all cases (R^2^ ≈ 0.67–0.77), indicating that liposomal systems do not behave as uniform matrices with purely Fickian diffusion [72].

In the case of CT-LT (liposomes with polymeric tannins), no antioxidant release was observed during the evaluation period, so no release curve was produced or kinetic fitting was conducted. This highlights their complete retention capacity, attributed to the high molecular weight of tannins and the size of their structures, contrasting with the passive release seen for small monomeric molecules such as epicatechin and gallic acid. Table 3 shows that, for the GA-LT, Ep-LT, and Cat-LT systems (gallic acid, epicatechin, and catechin encapsulated in LT), the release models fit the Korsmeyer–Peppas model with a high coefficient of determination (R^2^ > 0.97), with exponents n close to 0.7. This value indicates an anomalous release mechanism (non-Fickian transport), linked to a combination of diffusion and structural relaxation of the lipid bilayer [41,43,73]. The experimental data were also fitted to the Higuchi and Weibull models, following Equations (6), (7), and (8), respectively.

For the LTD formulations (GA-LTD and Ep-LTD), the Weibull model demonstrated the best fit (R^2^ > 0.98). For AG-LTD, the parameter β = 0.91 indicates a combined diffusion and relaxation mechanism. In contrast, Ep-LTD showed a β value of 0.07, which suggests a highly restricted and sub-diffusive release process [74].

The β-Weibull value of 0.07 observed in Ep-LTD suggests that epicatechin forms densely packed microdomains within the lipid bilayer, significantly limiting its mobility. This behavior aligns with molecular dynamics simulation observations showing stable aggregates formed through hydrogen bonding and hydrophobic interactions. The findings support the idea that polyphenols can stabilize the bilayer and alter its dynamic behavior, consistent with the effects seen in antioxidant-loaded liposomal systems [75]. It has been suggested that these interactions may cause a heterogeneous distribution of activation energies in the membrane, leading to sub-diffusive release kinetics [76]. This matches rheological results indicating increased rigidity of the lipid membrane in the presence of flavan-3-ol monomers. This effect can be attributed to the higher logP value of epicatechin, which promotes its retention within the bilayer and partially limits its diffusion into the external aqueous medium [77].

It is important to note that, although the Weibull model has been widely validated for drug release studies [78], it is an empirical model that does not necessarily provide detailed information on the molecular mechanisms involved. In this context, it is relevant to highlight that the LTD formulations also showed a good fit to the Korsmeyer–Peppas model (R^2^ > 0.90), which has a mechanistic basis. For AG-LTD, the n value was 0.714, while for Ep-LTD, it was 0.484, both of which are indicative of anomalous release (diffusion + relaxation), consistent with the behavior observed in LT formulations.

Overall, the results show that LT liposomal systems, especially LTD nanoliposomal systems, are very effective at encapsulating flavan-3-ols. The controlled release of less than 10% for LTD in aqueous simulant shows high retention of the active ingredient, combined with high structural stability and limited transport mechanisms, supports the potential of LT liposomal systems as functional and stable platforms for protecting and controlling the release of edible antioxidants, avoiding premature losses in food matrices and favoring their targeted release in later intestinal stages.

### 4.5. Discussion of Bioaccessibility and Digestion Release of Liposomal Suspensions

Simulated digestion of liposomal (LT) and nanoliposomal (LTD) systems revealed notable differences in release behavior, influenced by both the nature of the encapsulated compound and the structure of the liposomal system (Figure 6). In Figure 6a, bioaccessibility values exceeding 100% were observed for gallic acid (GA) and epicatechin (Ep) in the LT formulations (GA-LT and Ep-LT), indicating the possible release of additional phenolic structures. This may result from the partial breakdown of condensed tannins (CT) caused by the acidic and thermal conditions in the digestive environment [21].

Both liposomal formulations (LT and LTD) showed high bioaccessibility to small molecules such as gallic acid and epicatechin. Although the bioaccessibility of the main monomer epicatechin was 98.56 ± 0.81 for GA and 95.61 ± 0.58 for Ep in LTD, comparing both systems based on bioaccessible mass, the depolymerized nanoliposomal system (LTD) released a significantly greater amount of epicatechin (700 µg) compared to the LT system (30 µg) (Figure 6b), which can be expressed as a relative increase of 2333%, highlighting a major improvement in the release efficiency of the compound due to tannin depolymerization and the nanostructuring of the system.

Although bioaccessibility is a useful indicator of the fraction of a compound available for absorption, its actual bioavailability depends on additional factors such as intestinal permeability, presystemic metabolism, and interactions with the intestinal microbiota. In general, highly polymerized tannins with high molecular weight are not absorbed in the small intestine and rely on fragmentation and microbial metabolism in the colon [12]. In contrast, epicatechin exhibits high bioavailability, with urinary excretion studies indicating that approximately 95% is absorbed and enters systemic circulation [79]. Therefore, while it remains necessary to evaluate the in vivo bioavailability of the LTD system, the results obtained in vitro are highly promising.

The CT-LT formulation, on the other hand, showed no detectable release of condensed tannins by UHPLC. However, this lack of signal does not mean there is no release; instead, it could be due to the high reactivity of these polymers with proteins and digestive enzymes, forming stable covalent bonds that make their detection difficult with conventional analytical techniques [80]. Figure 7 illustrates the evolution of release over time during the digestion assay, monitored through a stepwise sampling scheme. It was observed that there was no significant release of compounds during the gastric phase, confirming the structural integrity of the lipid bilayer under acidic conditions [81,82].

Liposomes have been established as efficient systems for protecting and modulating nutrient release throughout the gastrointestinal tract. It has been shown that liposomal phospholipids can be enzymatically hydrolyzed to lysophospholipids, which are capable of forming micelles with bile salts, thus promoting more efficient intestinal absorption of nutrients [56]. In the case of resveratrol, it has been confirmed that liposomes maintain their structural integrity during the gastric phase, which significantly contributes to their protection against degradation [83]. Similarly, in liposomal formulations loaded with curcumin, the compact arrangement of the vesicle decreases the adsorption of gastric fluids on its surface by reducing the availability of binding sites, demonstrating that this organization improves the intestinal absorption of curcumin [84]. Furthermore, liposomes loaded with astaxanthin showed high stability under gastric conditions, which resulted in a significant increase in the bioaccessibility of this carotenoid [85].

This behavior aligns with previous studies suggesting that the release of liposome-encapsulated compounds mainly occurs during the duodenal phase. At this stage, the combined action of bile salts and digestive enzymes such as phospholipases encourages the gradual opening of the bilayer and subsequent release of polar compounds that are partially bound to the lipid matrix, while the oral and gastric phases proceed without significant release [86]. Consistent with the results of this study, Manca et al. (2020) [87] observed that liposomal formulations of grape seed extracts released phenolic compounds in a controlled and slow manner under simulated intestinal conditions. Although their systems included edge activators such as Tween 80 to enhance stability in acidic media, they reported partial release of polyphenols in duodenal environments, supporting the idea that polyphenol–bilayer interactions, combined with the viscosity and microstructure of the medium, influence restricted release pathways and unusual kinetics [87].

The increase in bioaccessibility of flavan-3-ols in LTD systems reached 2333% compared to LT. Although this value seems high, similar results have been reported in other nanostructured delivery systems. It has recently been reported that the bioaccessibility of liposome-encapsulated astaxanthin (39.61 ± 1.13%) was significantly higher than that of free astaxanthin (6.54 ± 1.42%) [88], while liposomal systems have been shown to exhibit an impressive release of encapsulated lycopene (1033.54 ± 0.32 μg/mL PS) and β-carotene (10.27 ± 0.02 μg/mL PS), significantly exceeding their bioaccessibility in free form [89]. These findings suggest that the observed magnitude is not isolated, but is consistent with the capacity of nanocarrier systems to transform bioactives into highly accessible fractions through size reduction, solubilization, and protection against irreversible interactions in the gastrointestinal tract.

The digestive stability of liposomes depends mainly on two factors: (i) the resistance of the lipid bilayer to disruption induced by pH variations, ions, and amphiphilic molecules present in the gastrointestinal environment, and (ii) the resistance to enzymatic degradation of the phospholipids that make up the membrane [84]. In this study, it was demonstrated that the developed formulations: (a) retained their structural integrity during prolonged storage (60 days), (b) maintained adequate colloidal stability reflected in a negative ζ potential, and (c) preserved their integrity under simulated gastric conditions, which translated into greater bioaccessibility of the encapsulated compounds. Together, these results confirm the formation of highly stable and functional liposomes, with adequate performance for applications aimed at improving the bioavailability of polyphenols in food matrices [83,84,85,90,91].

### 4.6. Applications and Future Perspectives

Liposome technology is fundamental in food and functional food applications. These applications include liquid formulations, such as dairy drinks, yogurts, juices, and soups [92], as well as solid formulations, such as meat, tofu, and cheese [37]. Furthermore, liposomes have been shown to maintain their stability against processes such as lyophilization, allowing their incorporation in powder form into flours and milks [24].

To promote further development and widespread use of these liposomal systems, it is necessary to implement efficient preparation approaches and safe formulations [90]. In this context, formulations designed as LTD have considered the use of GRAS solvents, including their reuse to reduce environmental impact, as well as clean and emerging methodologies, such as microwaves and ultrasound. This methodological strategy is promising for future industrial scaling.

## 5. Conclusions

Condensed tannins liposomal (LT) and depolymerized condensed tannins nanoliposomal (LTD) systems demonstrated excellent encapsulation capacity for flavan-3-ols, with LTD showing greater structural stability, nanometric structure, and thermal rigidity due to specific interactions between monomeric polyphenols and phospholipids.

Release studies showed controlled kinetics, with notable retention and restricted release of antioxidants in LTD, associated with the formation of microdomains in the lipid bilayer.

During simulated digestion, the lipid bilayer maintained its integrity under gastric conditions, releasing compounds mainly in the intestinal phase, where enzymatic action facilitated their release and full bioaccessibility. Nanostructuring and depolymerization of tannins in LTD significantly enhanced antioxidant release compared to conventional systems. These findings confirm that nanoliposomes with depolymerized polyphenols are an effective, stable, and functional platform for protecting and targeted releasing antioxidants, with great potential for nutraceutical and food applications.

## Figures and Tables

**Table 1:** Physicochemical characterization of initial grape seed condensed tannins (ST) and depolymerized grape seed condensed tannins suspensions (STD). Values correspond to the mean ± standard deviation (*n* = 3).

Characterization	ST	STD
TPC (GAeq mg/g)	241.7 ± 13.2 ^a^	287.4 ± 7.96 ^b^
Inhibition ABTS•+ (%)	89.53 ± 7.96 ^a^	90.47 ± 2.83 ^a^
Inhibition DPPH• (%)	88.71 ± 6.32 ^a^	89.62 ± 6.49 ^a^
Principal structure	Polymers with mDP * 8 ^a^	Epicatechin monomers ^b^
HPD (nm)	16,860 ± 1073 ^a^	6.2 ± 1.07 ^b^

^a,b^ Same letters in the same row indicate that there are no significant differences between means according to a multiple comparisons test (*p* < 0.05) and one-way ANOVA followed by Tukey’s post-hoc test (two-tailed, 95% CI). * mDP is the mean degree of polymerization.

**Table 2:** Characterization of liposomal suspensions of initial grape seed condensed tannins (LT) and depolymerized grape seed condensed tannins suspensions (LTD). Values correspond to the mean ± standard deviation (*n* = 3).

Parameter	LT	LTD
EE (%)	99.65 ± 0.01 *^,a^	83.11 ± 1.46 **^,b^
LC (%)	99.46 ± 0.01 *^,a^	97.24 ± 0.05 **^,a^
HPD (nm)	235.3 ± 21.4 ^a^	101.3 ± 7.8 ^b^
ζ (mV)	−56.0 ± 1.3 ^a^	−50.7 ± 1.1 ^b^
PDI	0.24 ± 0.10 ^a^	0.26 ± 0.13 ^a^

^a,b^ Same letters in the same row indicate that there are no significant differences between means according to a multiple comparisons test (*p* < 0.05) and one-way ANOVA followed by Tukey’s post-hoc test (two-tailed, 95% CI). These results are representative of the principal species in suspension CT* and Ep**.

**Table 3:** From the fitting of Equations (7)–(9) to the release data of liposomal and nanoliposomal antioxidants.

	Korsmeyer–Peppas			Higuchi		Weibull		
Molecule	R^2^	K_R_	*n*	R^2^	K_H_	R^2^	α	β
GA-LT	0.9979 *	0.247	0.763 ± 0.055	0.6781	0.233	0.9653	2.2 ± 0.1	0.91 ± 0.13
Cat-LT	0.9823 *	0.247	0.703 ± 0.032	0.7776	0.207	0.9767	6.6 ± 0.7	0.04 ± 0.01
Ep-LT	0.9738 *	0.268	0.656 ± 0.054	0.7518	0.208	0.9604	7.0 ± 1.9	0.04 ± 0.01
AG-LTD	0.9399	0.265	0.714 ± 0.056	0.7334	0.241	0.9863 *	3.7 ± 0.4	0.14 ± 0.02
Ep-LTD	0.9264	0.351	0.484 ± 0.027	0.7691	0.202	0.9903 *	5.8 ± 1.7	0.07 ± 0.02

* Indicates the most significant relationship coefficient (R^2^) for adjustments to different mathematical models of bioactive release.

## Data Availability

The original contributions presented in this study are included in the article/Supplementary Material. Further inquiries can be directed to the corresponding author.

## Supplementary Materials

The following supporting information can be downloaded at: https://www.mdpi.com/article/10.3390/antiox14091123/s1, Figure S1: Cumulative release graph over time for antioxidant species under release conditions at 37 °C and 170 rpm in food simulant D1, suspensions of condensed tannins (ST), depolymerized condensed tannins (STD), liposomal encapsulating condensed tannins (LT), and nanoliposomal encapsulating depolymerized condensed tannins (LTD); Figure S2: Cumulative concentration release (µg/mL) graph over time for antioxidant species under release conditions at 37°C and 170 rpm in food simulant D1, suspensions of liposomal encapsulating condensed tannins (LT), and nanoliposomal encapsulating depolymerized condensed tannins (LTD).

## Abbreviations

The following abbreviations are used in this manuscript:

PC	L-α-Lecithin Soybean with phosphatidylcholine purity ≥ 97%
ST	Condensed tannin suspensions
STD	Depolymerized condensed tannin suspensions
LT	Condensed tannins in liposome suspensions
LTD	Depolymerized condensed tannins nanoliposome suspensions
GA	Gallic acid
Ep	Epicatechin
Cat	Catechin
CT	Condensed tannins, polymeric fraction of ST
GA-LT	Gallic acid fraction encapsulated in LT
Cat-LT	Catechin fraction encapsulated in LT
Ep-LT	Epicatechin fraction encapsulated in LT
CT-LT	Condensed tannins fraction encapsulated in LT
GA-LTD	Gallic acid fraction encapsulated in LTD
Ep-LTD	Epicatechin fraction encapsulated in LTD
