# Supplementary material for: Depolymerization and Nanoliposomal Encapsulation of Grape Seed Condensed Tannins: Physicochemical Characterization, Stability, In Vitro Release and Bioaccessibility
Source: Antioxidants (Basel). 2025 Sep 16;14(9):1123. doi: 10.3390/antiox14091123 (PMC12466359; doi:10.3390/antiox14091123)
Supplement: Supplementary file 1 [file antioxidants-14-01123-s001.zip › Figure S2.pdf]

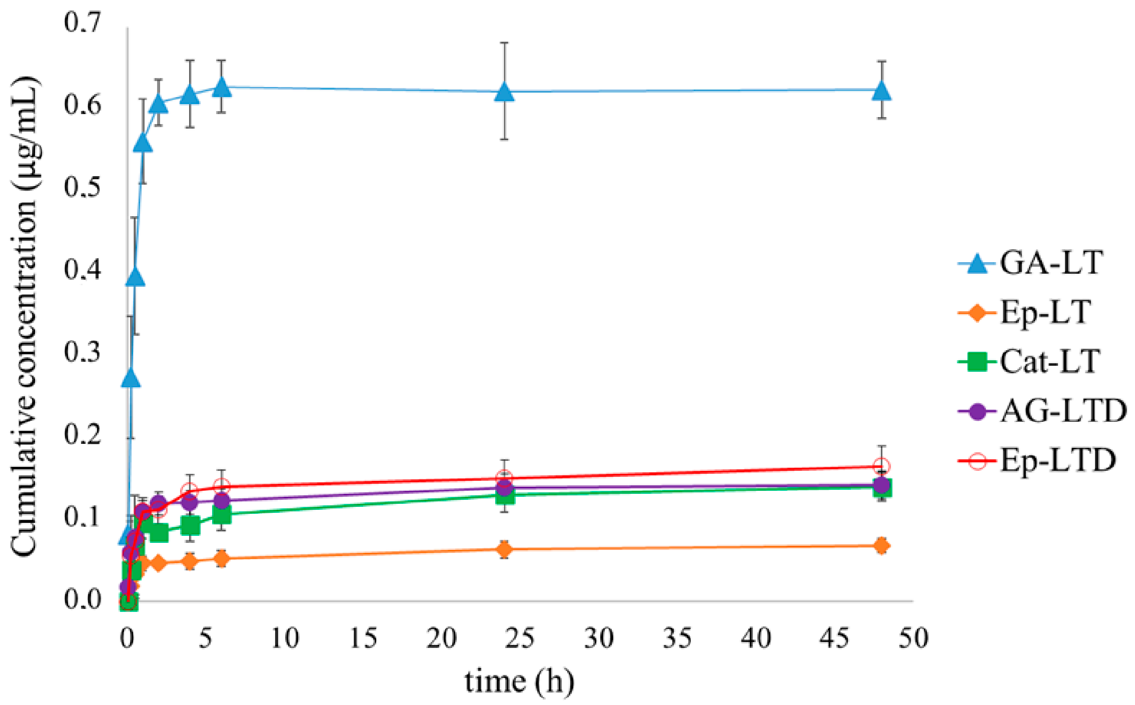

Figure S2. Cumulative concentration release (µg/mL) graph over time for antioxidant species under release conditions at 37°C and 170 rpm in food simulant D1, suspensions of liposomal encapsulating condensed tannins (LT), and nanoliposomal encapsulating depolymerized condensed tannins (LTD).
